# Temporal Course of 2014 Ebola Virus Disease (EVD) Outbreak in West Africa Elucidated through Morbidity and Mortality Data: A Tale of Three Countries

**DOI:** 10.1371/journal.pone.0140810

**Published:** 2015-11-11

**Authors:** Ying-Hen Hsieh

**Affiliations:** Department of Public Health and Center for Infectious Disease Education and Research, China Medical University, Taichung, 40402, Taiwan; Metabiota, UNITED STATES

## Abstract

The explosive outbreak of Ebola virus disease (EVD) in West Africa in 2014 appeared to have lessened in 2015, but potentially continues be a global public health threat. A simple mathematical model, the Richards model, is utilized to gauge the temporal variability in the spread of the Ebola virus disease (EVD) in West Africa in terms of its reproduction number *R* and its temporal changes via detection of epidemic waves and turning points during the 2014 outbreaks in the three most severely affected countries; namely, Guinea, Liberia, and Sierra Leone. The results reveal multiple waves of infection in each of these three countries, of varying lengths from a little more than one week to more than one month. All three countries exhibit marginally fluctuating reproduction numbers during June-October before gradually declining. Although high mobility continues between neighboring populations of these countries across the borders, outbreak in these three countries exhibits decidedly different temporal patterns. Guinea had the most waves but maintained consistently low transmissibility and hence has the smallest number of reported cases. Liberia had highest level of transmission before October, but has remained low since, with no detectable wave after the New Year. Sierra Leone has gradually declining waves since October, but still generated detectable waves up to mid-March 2015, and hence has cumulated the largest number of cases—exceeding that of Guinea and Liberia combined. Analysis indicates that, despite massive amount of international relief and intervention efforts, the outbreak is persisting in these regions in waves, albeit more sparsely and at a much lower level since the beginning of 2015.

## Introduction

An explosive outbreak of Ebola virus disease (EVD) has occurred in Guinea, West Africa since March 2014. By August, the World Health Organization (WHO) declared the outbreak to be a “public health emergency of international concern” [1]. The extensive international attention that followed has not helped to lessen its catastrophic force in three countries (Guinea, Liberia, and Sierra Leone) that suffer the most. By April 16 2015, WHO reported 25831 confirmed, probable, or suspected cases in these three countries with 10699 deaths [2]. Local infections of EVD have occurred outside of Africa, e.g., in the United States of America, United Kingdom, and Spain, offering indication of its potential for global spread with high mortality rate [3, 4]. Although EVD was first identified in 1976 in Democratic Republic of Congo (DRC) and has emerged in several countries in the central African region (e.g., DRC, Sudan, Uganda) in the years since, the 2014 West Africa EVD outbreak was by far the most devastating of all, for reasons yet to be established.

To ascertain the full threatening potential of the epidemic, many modeling studies have reported various estimates and projections, by using a wide range of quantitative approaches and obtaining estimates of transmission potential in the form of the reproduction number [4–16]. All of these studies were carried out in October or earlier, using Ebola data mainly from July to October. Several of the studies (e.g., [4–5, 15]) published in late September attempted to quantify the temporal variations in reproduction number, but with differing results. Some reported early signs of a decrease in transmission as revealed by estimated effective reproduction number [15].

Given the volatilely unstable nature of the outbreaks in these countries under constantly changing circumstances, it is important to capture the temporal changes and current trending in the modeling. In this work, a simple mathematical model and EVD case data from Guinea, Liberia, and Sierra Leone is utilized to fit the Ebola data during various time intervals from June to late December, in order to pinpoint the occurrence of waves of reported cases and to trace through the temporal changes of the epidemic in these three countries during the recent months, in the form of the upturn and downturn of disease incidence during each wave. The results enable us to elucidate the temporal variations in transmission potential of the Ebola virus.

## Methods

### Data

Due to data collection issues, the data used is subject to changes due to ongoing reclassification, retrospective investigation and availability of laboratory results [17]. To eliminate artificial variations in data caused by diagnosis/reporting issues, the suspected cases reported in the WHO reports are deleted as many other studies had done (e.g., [4–5, 7]), to inaccuracy in the reporting of cases. Subsequently, the combined number of reported confirmed/probably case and death data up to March 17/18, 2015 for Guinea, Liberia, and Sierra Leone as made available by WHO in WHO Situation Summary [2] on March 20, 2015 are used. The time series of combined total case number of these three countries up to September 28 is also fitted to the Richards model, since it was the last date that synchronized case reporting from the three countries was available.

### Richards Model

The Richards model [18] is a simple mathematical model of the form

$$C(t)\;=\;K{\left[1+e^{-ra(t-t_{i}-(\text{ln}\;a)/ra)}\right]}^{-1/a}.$$

In the context of infectious disease modeling [19–20], *C*(*t*) is the cumulative number of reported cases of infections (or any other quantity of disease morbidity/mortality such as number of deaths, hospitalizations, etc., see [21]) at week *t*. *K* is the final case number over a single wave of outbreak, *r* is the per capita growth rate of the cumulative case number, *a* is the exponent of deviation of the cumulative case curve, and *t*_*i*_ is a turning point of the epidemic (which signifies the moment of upturn or downturn for the increase in the cumulative case number).

The basic premise of the Richards model is that the incidence curve of a single wave of cases consists of a single peak of high incidence which starts initially with exponential growth followed by saturation, resulting in an S-shaped *cumulative* case curve and a single turning point of the outbreak. This turning point *t*_*i*_, which is defined to be the point in time at which the rate of accumulation changes from increasing to decreasing, or vice versa, can be easily pinpointed via fitting the Richards model to the cumulative data in question.

When more than one wave of infections occurs, a variation of the S-shaped Richards model was proposed [20], which distinguishes two types of turning points. In addition to the above-mentioned turning point signifying the end of exponential growth, a second type of turning point is present in a multi-wave epidemic where the growth rate of the cumulative case number begins to increase again, signifying the beginning of the next wave. For further illustrations, the readers are referred to [20, 22], where the incidence curves for the 2003 Great Toronto Area (GTA) SARS and the 2007 Taiwan dengue outbreaks containing two peaks (or two turning points of the first type) and one valley (or a turning point of second type) are investigated.

### Reproduction Number

For the basic reproduction number *R*_0_, the formula *R*_0_ = *exp*(*rT*) is employed, where *T* is the generation interval of the disease or the average interval from onset of one individual to the onset of his/her contacts. It has been shown mathematically [23] that, given a growth rate *r*, the expression *R*_0_ = *exp*(*rT*) provides an upper bound for basic reproduction number, regardless of the assumed distribution of the generation interval *T*. Please note however, that since the aim is to fit EVD time series data from various time periods during the epidemic, the estimate obtained is not the *basic* reproduction number, but the *effective* reproduction number *R* of the fitted time period.

The model parameters of epidemiological importance are *K*, *r*, and the turning point *t*_*i*_ of the epidemic. The cumulative epidemic data can be fitted to the Richards model to obtain estimates of these model parameters, using any standard software with least-squares approximation tool, e.g., SAS, Matlab, etc. More applications of the Richards model on other infectious disease outbreaks such as dengue, influenza, HIV also can be found respectively in [22, 24–25].

## Results

**Fig 1:**
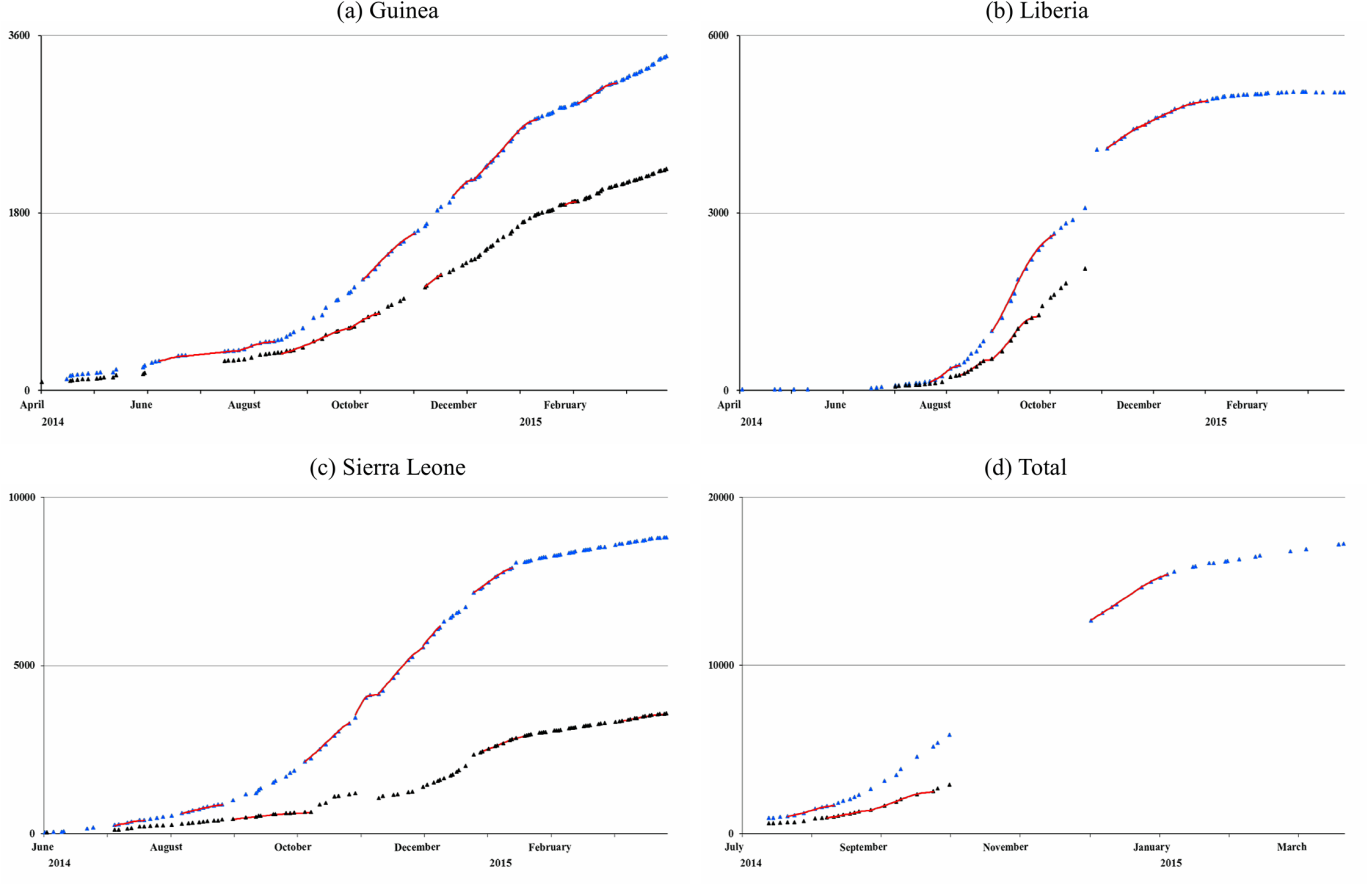
Richards model fit for each wave of 2014 EVD outbreak data in (a) Guinea, (b) Liberia, (c) Sierra Leone, and (d) total. For each figure, black dot denotes reported death data, blue dot denotes reported confirmed/probable case data, red curve denotes model-fitted number of deaths, and green curve denotes model-fitted confirmed/probably case number.

The Richards model is fitted to the cumulative confirmed/probably case and death data at different time intervals for each country as well as for the combined total numbers, and was able to detect multiple waves for each country. Details of the data fitting procedure are provided in S1 File. By fitting the EVD case and death data of Guinea, Liberia, and Sierra Leone to the Richards model (Fig 1), one is able to detect seven small waves from case data and four waves from death data during June 5 to February 18, 2015 in Guinea. For Liberia, four waves of cases and two waves of deaths between July 20 and December 28 are pinpointed. For Sierra Leon seven waves of cases and three waves of deaths during June 30 to March 15, 2015 are detected. By combining the total case number of the three countries, two waves of cases and two waves of deaths from July 20 to December 31 have been obtained. The resulting time intervals in which the Richards model is fitted, and the corresponding turning points and reproduction numbers with the 95% confidence interval (CI) from model fitting, are summarized in Table 1, with *R* computed using a generation time of *T* = 15.3±9.3 [3].

**Table 1:** Summary table for time intervals of Richards model fit, data fitted, and estimates of the turning point and reproduction number with 95% confidence intervals in parenthesis.

Country	Data	Time interval	*t*_*i*_	Turning point	Reproduction number *R*
**Guinea**	case	6/5~6/20	7.56 (6.94,8.18)	6/13	1.24 (1.08,1.41)
		6/20~7/20	17.47 (12.38,22.56)	7/8	1.08 (1.03,1.14)
		7/20~8/9	5.25 (1.01,9.48)	7/26	1.28 (1.08,1.48)
		9/28~10/27	7.30 (1.44,13.15)	10/6	1.31 (1.08,1.54)
		11/18~11/30	2.62 (0.98,4.26)	11/21	1.17 (1.06, 1.28)
		11/30~1/4	18.62 (15.78,21.46)	12/19	1.14 (1.05,1.23)
		1/27~2/18	10.03 (3.78,16.27)	2/7	1.06 (1.02,1.10)
	death	8/13~9/21	26.20 (21.72,30.69)	9/9	1.27 (1.09,1.46)
		9/21~10/7	5.13 (1.79,8.47)	9/27	1.31 (1.09,1.52)
		11/2~11/11	4.56 (3.14,5.98)	11/7	1.23 (1.07,1.38)
		1/20~1/26	2.27 (1.61,2.92)	1/23	1.05 (1.02,1.09)
**Liberia**	case	7/20~8/6	10.71 (9.21,12.21)	7/31	3.58 (0.76,6.40)
		8/25~9/30	13.28 (10.84,15.72)	9/8	2.16 (1.08,3.24)
		10/31~11/22	8.60 (3.56,13.64)	11/9	1.08 (1.03,1.13)
		11/15~12/28	10.53 (5.08,15.98)	11/26	1.05 (1.02,1.08)
	death	8/6~8/25	11.98 (10.19,13.77)	8/18	2.22 (1.14,3.30)
		8/25~9/21	13.35 (11.71,14.99)	9/8	2.09 (1.14,3.03)
**Sierra Leone**	case	6/30~7/14	6.71 (5.49,7.93)	7/7	2.12 (1.14,3.09)
		8/1~8/20	9.38 (6.37,12.40)	8/11	1.43 (1.12,1.75)
		9/28~10/19	12.46 (10.48,14.44)	10/11	1.44 (1.12,1.76)
		10/22~11/2	2.17 (1.17,3.16)	10/25	1.70 (1.15, 2.25)
		11/2~11/23	7.79 (4.59,11.00)	11/10	1.30 (1.09,1.51)
		11/23~12/1	3.47 (2.79,4.15)	11/27	1.24 (1.08,1.39)
		12/17~1/4	6.23 (1.74,10.72)	12/24	1.10 (1.04,1.17)
	death	8/25~10/1	9.56 (5.21,13.90)	9/4	1.28 (1.08,1.48)
		12/20~1/12	9.68 (7.90,11.45)	12/30	1.17 (1.06,1.28)
		2/24~3/15	9.82 (6.01,13.63)	3/6	1.06 (1.02,1.09)
**Total**	case	7/20~8/9	11.40 (9.06,13.73)	8/1	1.62 (1.14,2.10)
		11/28~12/31	17.17 (12.84,21.50)	12/16	1.11 (1.04,1.18)
	death	8/6~8/25	12.21 (10.00,14.42)	8/19	1.48 (1.13,1.83)
		8/25~9/21	13.06 (10.94,15.18)	9/8	1.58 (1.14,2.02)

**Fig 2:**
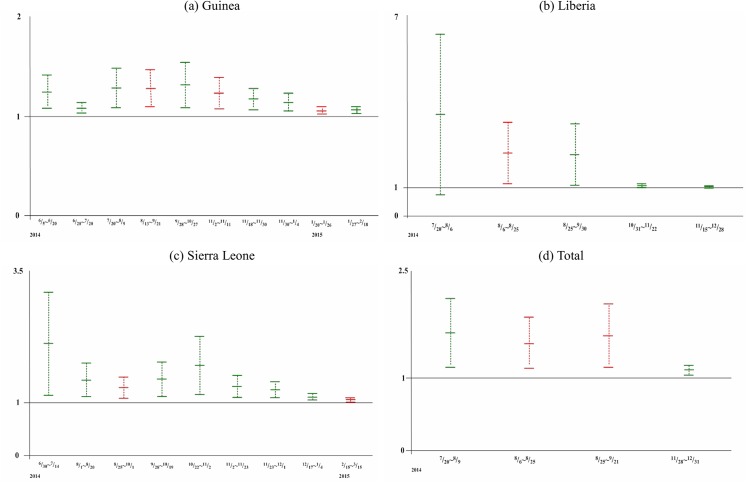
Reproduction number *R* and its corresponding 95% CI for each wave in (a) Guinea, (b) Liberia, (c) Sierra Leone, and (d) total. The middle bar denotes the mean estimate for *R*, while the upper and lower bars denote the upper and lower bounds for 95% CI. Green denotes estimates using the confirmed/probable case data, and red denotes estimates using the number of deaths.

**Fig 3:**
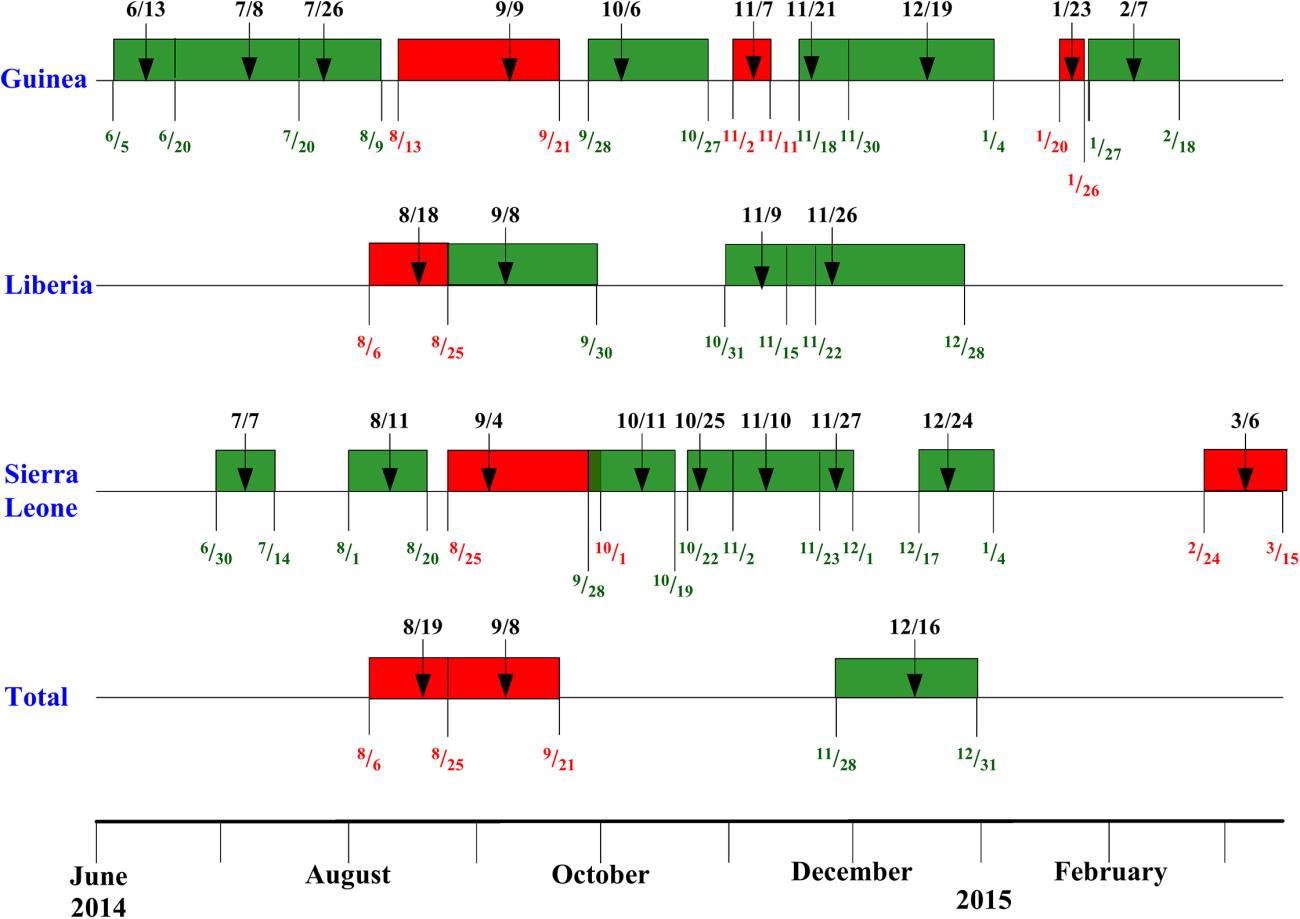
Chronological timelines for the waves of 2014 EVD outbreak between June 5 2014 to March 15 2015 in (a) Guinea, (b) Liberia, (c) Sierra Leone, and (d) total. Green bar denotes a wave of confirmed/probably case number and Red bar denotes a wave of deaths.

To further illustrate the temporal changes of transmission potential of Ebola virus in each country since June, Fig 2 shows the time periods in which a reproduction number *R* was obtain. Here the data used to obtain the estimate was shown in color, with green for case number and red for number of deaths. To further highlight the temporal progression of the outbreak in each country, chronological timelines of the waves were provided in Fig 3.

## Discussion

### Morbidity and mortality data

Due to data collection issues, the data used is subject to changes due to ongoing reclassification, retrospective investigation and availability of laboratory results [17] which often occurs during the course of an epidemic outbreak [26]. In attempt to eliminate artificial variations in data caused by diagnosis/reporting issues, the subsequent deletion of suspected cases reported in the WHO reports (e.g., [4–5, 7]) might led to an underreporting of cases, in addition to many more unreported or asymptomatic cases. More precisely, the waves that were pinpointed through modeling are waves of reported cases/deaths, which does not necessarily correspond exactly to waves of infections, but nonetheless give indication to the variations in the temporal course of the epidemic that had occurred. Therefore, the results need to be interpreted qualitatively rather than quantitatively, in the sense that the relative temporally variation in the reproduction number is more revealing than the actual magnitude.

On the other hand, if these artificial diagnosis/reporting issues remain consistent throughout the epidemic, any underreporting that had indeed occurred would not have too much actual impact on the detection of waves or the timing of the turning points. Furthermore, note that while previous modeling studies of infectious disease outbreaks in literature have been shown that multiple estimates can be obtained from distinct epidemic data (case, hospitalization, and deaths), the estimates of reproduction number are often comparable [21, 26–27].

However, clinical progression of an infected person from diagnosis/reporting to death/recovery dictates that a wave detected using case data (in green color in Fig 3) must temporally precede the same wave detected through death data (in red color). Some of these waves in Table 1 are redundant. That is, since death is a consequence of a reported case after a time lag, some waves of reported deaths that were capture by the Richards model were in fact the same waves that had previously been detected from the reported case data, after a lag of average time from reporting to death. For a case in point, the wave of cases in Sierra Leone during December 17-January 4, 2015 is clearly the same wave that results in reported deaths during December 20-January 12, 2015. Hence, these redundant waves are deleted from Figs 2 and 3, and the discussions, in order to elucidate what the results truly inform us.

### Country-specific transmissibility of EVD

The initial waves for Liberia and Sierra Leone exhibit significantly wider 95% CIs comparing to latter waves of these two country, perhaps an indication of the volatility and uncertainty surrounding case diagnosis/reporting during the early phase of the outbreak in July. Hence we discard these two early waves. Subsequently, the reproduction number ranges between 1.05–1.31 for Guinea, between 1.05–2.22 in Liberia, between 1.06–1.70 in Sierra Leone, and between 1.11–1.62 for the combined total of the three countries.

Both Guinea and Sierra Leone data generated multiple waves that remain at a low level of transmissibility with mean reproduction number between 1 and 2. However, the lower bound of reproduction number during this initial wave (1.14) is consistently similar to the lower bounds of subsequent waves detected in Sierra Leone, and hence might have been caused by greater uncertainty in diagnosis/reporting during the initial stage of epidemic, rather than being a true indication of greater transmissibility.

Interestingly, the three waves for combined total numbers of cases and deaths coincide with three of the four waves detected using the case and death data for Liberia (see Fig 3), with the lone exception occurring when the data for combined total was unavailable in November. This gives indication that the epidemic in these three countries in West Africa basically follows a similar temporal pattern as that of Liberia. However, both the numbers of reported cases and deaths in Liberia are actually smaller than those of Sierra Leone, showing that the country with the largest outbreak (Sierra Leone) does not necessarily dictates the overall temporal changes of the epidemic in that region.

From Table 1 and Fig 2, the transmission potential of EVD in all three countries, as quantified by the estimated reproduction number *R*, fluctuated slightly but remained relatively consistent during all waves of the epidemic for Guinea and Sierra Leone, but only from August to September in Liberia. The observed variations in range of 95% CIs is mainly due to the varying lengths of the waves resulting and thus the number of data points for the model fit. The reproduction number for Liberia decreased significantly after November, an indication of improved situation for Liberia.

### Reproduction number for 2014 West Africa EVD

To compare the results of this study with previous modeling studies, Table 2 lists results on estimates of reproduction number for 2014 West Africa Ebola outbreak. The date of publication/online is given as an indication of the timeline of the data that is available at the time when these studies were carried out. The results for the time periods before October (row 1 in Table 2) are in general agreement with the other estimates in the literature. Estimate for Liberia tends to be higher than most other studies, while estimates are somewhat lower for Guinea and Sierra Leone as well as the combined total number. However, estimates for Liberia for August and September (mean *R*: 2.09–2.22) is close to estimates for Montserrado County, Liberia during June 4 to September 23 using a transmission model (2.49 in [11]) and for Liberia in August using a logistic growth model (2.4 in [15]), corroborating the results of this study that the transmissibility of EVD was at its highest during August-September in Liberia. This study also finds that the reproduction numbers in all countries declined after September, corroborated by a recent modeling study using district-level data in Sierra Leone found that the reproduction number in all districts declined between August and December [28].

**Table 2:** Summary table of recent literature on reproduction number for 2014 Ebola (EVD) outbreak in West Africa, with online publication date of each article in parenthesis. Note that for this study, multiple data periods are used for each country.

Article (date)	Estimate	Guinea	Liberia	Sierra Leone	Total
**This study**	*R* (before 10/31)	1.08–1.31	2.09–2.22	1.28–1.70	1.48–1.62
	*R* (after 11/1)	1.05–1.23	1.05–1.08	1.10–1.30	1.11
Althaus [9] (9/2)	*R*_0_	1.51 (1.50,1.52)	1.59 (1.57,1.60)	2.53 (2.41,2.67)	-
Gomes et al. [8] (9/2)	*R*_0_ (overall)	-	-	-	1.8 (1.5,2.0)
	*R*_0_ (SEIR)	-	-	-	2.1 b(1.9,2.4)
Fisman et al. [6] (9/8)	*R*_0_	-	-	-	1.6–2.0
Nishiura and Chowell [7] (9/11)	*R*_t_	1.0	1.4–1.7	1.4–1.7	-
Towers et al. [5] (9/23)	*R*	1.9 (1.6,2.3)	1.8 (1.6,2.0)	1.2 (1.0,1.50	1.6 (1.4,1.8)
WHO Ebola Response Team [3] (9/23)	Initial *R*_0_	1.71 (1.44,2.01)	1.83 (1.72,1.94)	2.02 (1.79,2.26)	-
	Current *R*	1.81 (1.60,2.03)	1.51 (1.41,1.60)	1.38 (1.27,1.51)	-
Stadler et al. [10] (10/6)	*R*_0_ (medium)	-	-	-	2.18 (1.24,3.55)
Lewnard [11] (10/24)	*R*_0_	-	2.49 (2.38–2.60)	-	-
Yamin et al. [12] (10/28)	*R*_0_	-	1.73 (1.66,1.83)	-	-
Pandey et al. [13] (10/30)	*R*_0_	-	1.63 (1.59,1.66)	-	-
Rivers et al. [14] (11/6)	*R*_0_	-	-	-	2.22*
Chowell et al [15] (11/20)	*R*_0_			2.4	
	*R*	1.4	1.2	1.3–1.7	
Webb et al. [16] (1/30)	*R*_0_		1.54	1.26	

*Reported data including suspected cases.

There does not seem to be a significant change in the ranges of reproduction numbers during the time period in any of the three countries. Although in comparison, Guinea has the smallest, between 1.05–1.31, while Liberia has the largest reproduction number with mean estimate between 2.09–2.22 in August-September but significantly lower reproduction number (1.05–1.08) after November. Sierra Leone has most reported cases and deaths, but does not have a significantly larger reproduction number than other 2 countries, ranging between 1.06–1.70 after August, with the low being most recent wave in February-March, 2015. The mean reproduction number for three countries combined total is range between 1.48–1.62.

Temporally, although each country exhibits different pattern in its transmissibility (see Fig 2), reproduction numbers for each country fluctuate without any noticeable trend before November, likely an indication of stochastic variations that existed in reporting of earlier cases in each country. Mover, Sierra Leone has the most cases among the 3 countries, although its reproduction number was lower than that of Liberia until after November. Therefore, although reproduction number provides an indication of transmission potential of an infectious disease in a particular community during a particular time period (wave), one should not simply equate its magnitude with actual outbreak severity or duration of the wave.

Moreover, it has been proposed in earlier studies (e.g., [28]) that any outbreak is in fact a single realization of an underlying stochastic process that could exhibit considerable variability among repeated realizations, and hence the same stochastic process produces a range of realizations that each yields a different R_0_ estimate. The different R_0_ estimates listed in Table 2 are the results of different estimation procedures using data from a single realization of a stochastic process. For a lucid discussion on reliability of model fit with regard to West Africa ebola data, the readers are referred to [28].

### Country-level vs. local

The present study, similar to most other modeling studies on West Africa EVD outbreak, make use of the national case data, although some studies using local data have yielded similar results on reproduction number (e.g., [11], [29]). A recent study using national, subnational, and regional data [30] has found significant differences in the growth patterns, perhaps due to local differences in important factors such as reporting, behavior change, impact of intervention, etc., factors that tend to be suppressed when combining them in a national total. The epidemic data from these three countries clearly reflect a complex spatial structure, i.e., spatial spread of the disease across neighboring regions of these three countries, as pointed out in several studies (e.g., [28, 31]). Although modeling complex spatial structure often requires incorporating spatial structure in model construction and, more importantly, information on spatial movement of the populations across different regions, our multi-wave results and aggregated estimates for R_0_ using aggregated country-specific and combined total data highlight the nature of spatial heterogeneity that had existed during the epidemic.

In this study, by combining the national totals, the temporal course of the West Africa outbreak (as quantified by waves and turning points detected via the Richards model) tends to follow a similar pattern as that of Liberia, although Liberia did not have the most reported cases among the three countries. On the other hand, the transmission potential of EVD (as quantified by reproduction number *R*) estimated from the combined total case number of the three countries (range of mean *R*: 1.48–1.62) appears to fall consistently between the estimates for Liberia (which tends to have comparatively higher *R*) and the other two countries, whenever the waves in these countries coincide, until December when mean estimates of *R* for all countries as well as the combined total are around 1.10 with narrow 95% CIs, perhaps the result of some synchronization of local control and international relief taking efforts.

In summary, the outbreak in these three countries exhibits decidedly different temporal patterns, despite high mobility among the populations. Guinea has had the most waves but maintained a low level of transmission, and hence has smallest number of reported cases and deaths. Liberia had highest level of transmission before October, but has remained at low level since then and no detectable wave since the New Year. Sierra Leone had gradually declining waves since October, but still generated detectable waves up to March 2015, and hence has cumulated the largest number of cases, exceeding that of Guinea and Liberia combined since end of the year. The EVD epidemic is still persisting in waves in West Africa, albeit more sparsely and at a much lower level since the beginning of 2015.

The main limitation for this study is the data quality, due to data collection issues by local health authority, including: reporting of numbers subject to change due to ongoing reclassification, retrospective investigation and availability of laboratory results; data not reported due to the high proportion of probable and suspected cases that are reclassified; data not being available; and data missing for some dates in some countries [17]. Some of these issues are common in infectious disease outbreak data collection and real-time modeling [26]; although for the current Ebola epidemic in West Africa the problem is magnified due to instability in the region.

## Supporting Information

S1 File (DOCX)
