# Supplementary material for: Temporal Course of 2014 Ebola Virus Disease (EVD) Outbreak in West Africa Elucidated through Morbidity and Mortality Data: A Tale of Three Countries
Source: PLoS One. 2015 Nov 11;10(11):e0140810. doi: 10.1371/journal.pone.0140810 (PMC4641704; doi:10.1371/journal.pone.0140810)
Supplement: S1 File — (DOCX) [file pone.0140810.s001.docx]

**S1: Description of statistical data fitting of the Richards model**

The cumulative confirmed/probably case and death data is fitted separately to the Richards model to obtain estimates of *K*, *r*, and *t_i_*, using NLIN subroutine in SAS, a nonlinear least-squared approximation routine. We start the data fitting from the beginning of the outbreak for each country by testing time interval of various lengths for its goodness of fit to the Richards model, signifying a wave of cases of deaths. Here we use the adjusted $R^{2}$ [1] as the measure of goodness of fit to determine the exact time interval for each wave. The adjusted $R^{2}$ is defined as

$Adj\text{ }R^{2}=1-\frac{SSe/df_{e}}{SSt/df_{t}}$,

where $SSe$ is the sum of squares of residuals, $SSt$ is the total sum of squares, $df_{e}$ is the degrees of freedom of $SSe$, and $df_{t}$ is the degrees of freedom of $SSt$.

We continue with the fitting until a time period with the best adjusted $R^{2}$ measure of goodness of fit to the Richards model is found, signifying a wave. We then start at its end point and proceed to locate the next wave in similar manner. The procedure is repeated until we reach the end of the data time period, thus locating all waves that had occurred. More precisely, we determine the successive time intervals during which the Richards model will give the best fit for a wave of cases of deaths that had occurred.

In the Table below, we provide the results of adjusted $R^{2}$ measure of goodness of fit for the time intervals determined to have the best adjusted $R^{2}$ measure of goodness of fit for each wave of cases or deaths detected for the West Africa Ebola epidemic

**References**

1. Theil H. Economic Forecasting and Policy. Amsterdam: North Holland; 1961.

Table. The adjusted $R^{2}$ measure of goodness of fit for each wave detected for the West Africa Ebola epidemic.

| **Country** | **Data** | **Time interval** | **Adjusted** $R^{2}$ |  |
| --- | --- | --- | --- | --- |
| **Guinea** | case | 6/5~6/20 | 0.9999 | |
|  |  | 6/20~7/20 | 0.9999 | |
|  |  | 7/20~8/9 | 0.9999 | |
|  |  | 9/28~10/27 | 0.9999 | |
|  |  | 11/18~11/30 | 0.9999 | |
|  |  | 11/30~1/4 | 0.9999 | |
|  |  | 1/27~2/18 | 0.9999 | |
|  | death | 8/13~9/21 | 0.9998 | |
|  |  | 9/21~10/7 | 0.9999 | |
|  |  | 11/2~11/11 | 0.9999 | |
|  |  | 1/20~1/26 | 0.9999 | |
| **Liberia** | case | 7/20~8/6 | 0.9993 | |
|  |  | 8/25~9/30 | 0.9997 | |
|  |  | 10/31~11/22 | 0.9999 | |
|  |  | 11/15~12/28 | 0.9999 | |
|  | death | 8/6~8/25 | 0.9997 | |
|  |  | 8/25~9/21 | 0.9997 | |
| **Sierra Leone** | case | 6/30~7/14 | 0.9999 | |
|  |  | 8/1~8/20 | 0.9999 | |
|  |  | 9/28~10/19 | 0.9999 | |
|  |  | 10/22~11/2 | 0.9999 | |
|  |  | 11/2~11/23 | 0.9999 | |
|  |  | 11/23~12/1 | 0.9999 | |
|  |  | 12/17~1/4 | 0.9999 | |
|  | death | 8/25~10/1 | 0.9999 | |
|  |  | 12/20~1/12 | 0.9999 | |
|  |  | 2/24~3/15 | 0.9984 | |
| **Total** | case | 7/20~8/9 | 0.9999 | |
|  |  | 11/28~12/31 | 0.9999 | |
|  | death | 8/6~8/25 | 0.9999 | |
|  |  | 8/25~9/21 | 0.9998 | |
